# A Comparative Study on the Effects of Three Nano-Materials on the Properties of Cement-Based Composites

**DOI:** 10.3390/ma13040857

**Published:** 2020-02-13

**Authors:** Chao Fu, Chunyan Xie, Jing Liu, Xiuli Wei, Dake Wu

**Affiliations:** 1 College of Engineering and Technology, Southwest University, Chongqing 400715, China; fcswu2018@163.com (C.F.); swauyan@swu.edu.cn (C.X.); jingliu97@163.com (J.L.); 2 Chongqing Academy of Agricultural Sciences, Chongqing 401329, China; weixiuli.cau@126.com

**Keywords:** nanomaterials, cement-based composites, mechanical properties, strength benefit

## Abstract

The application of nano-materials to modify construction materials has become a research hotspot in recent years, but often different scholars use different research methods and reach different conclusions about the same material, which is not conducive to the performance comparison between different materials. In this paper, nano-SiO_2_, carbon nanotubes (CNTs) and nanocrystalline cellulose (NCC) were used as raw materials to prepare cement-based composites to compare the effects of the three nanomaterials on the mechanical and water absorption properties under the same experimental conditions, and their principles were investigated via The scanning electron microscope (SEM), X-Ray Diffraction (XRD) and other microscopic analysis testing methods. At the same time, strength benefit indexes are introduced to comprehensively evaluate the economics of the strength improvement provided by the three kinds of nanomaterial. The results show that doping with nano-SiO_2_, CNTs and NCC can promote the hydration process of cement effectively. The composite material exhibits excellent mechanical properties at the macro level because of the nucleation and filling effect of nano-SiO_2_, and the bridging and strengthening effects of CNTs and NCC. The compressive strength increased by 45.13%, 28.31% and 44.19% at 7d, and 23.09%, 18.40% and 23.40% at 28d. The flexural strength of 7d increased by 31.00%, 36.22 and 54.81%, and 14.91%, 22.23% and 30.46% at 28d. The water absorption is SiO_2_ < NCC < CNTs, and the nano-SiO_2_ is lower than the other two materials at least 15.54%. CNTs group has the lowest compressive strength benefit, which is 16.91 yuan/m^3^, and the lowest flexural strength benefit is NCC, which is 3.59 yuan/m^3^.

## 1. Introduction

Cement-based materials are the foundational materials of civil engineering construction in modern society, and also some of the most widely used materials in the civil engineering field. However, with the expansion of application scope and increasingly harsh environment, traditional cement-based materials have failed to meet the high performance requirements in special occasions such as earthquakes and the complex temperature stress of mass concrete due to the low toughness, low bending and tensile strength [1]. As a result, the research and application of new cement-based composite materials have become a new hot field of engineering applications.

There are two main methods for the preparation of new cement-based composite materials: one of them is adding a certain amount of macro-sized fibers such as steel fiber, carbon fiber, polypropylene fiber and glass fiber to traditional cement-based materials, which is a commonly used approach today [2,3,4]; these substances mainly improve the mechanical properties of cement-based materials by changing the stress transmission path, bridging, preventing crevices and strengthening of fiber materials. However, this can only improve the mechanical properties of materials on the macroscopic scale, and it is powerless on the microscopic scale, such as nanoscale defects, so the second method emerged, which is the addition of nanomaterials. Due to their macroscopic morphology, nanomaterials have surface effects, size effects, quantum effects and volume effects that macroscopic objects do not have, which can significantly increase the nucleation sites of cement hydration products. By bridging and paving the way in the micropores of test pieces, the hydrated product is formed into a whole net-like structure earlier and faster, to increase the strength [5,6].

At present, the application research on various nanomaterials in cement-based composites is mainly about the properties of single nanomaterials. Kawashima et al. [7] studied the hydration heat release process, compressive strength and flexural strength of cement paste with nano-CaCO_3_ for 24 h. The results showed that the incorporation of nano-CaCO_3_ could accelerate the pace of cement hydration and increase the compressive strength of cement-based materials at various ages. Jo et al. [8] found that nano-SiO_2_ could improve the compressive and flexural strength of mortar significantly by filling the microstructure. Madandoust et al. [9] compared the different performance properties of nano-SiO_2_, nano-Fe_2_O_3_ and nano-CuO, such as the strength, water absorption and electrical resistivity, etc. They found that the effect of nano-CuO on mortar strength is the most obvious among the three nanomaterials, the effect of nano-SiO_2_ is close to that of nano-CuO, and nano-Fe_2_O_3_ has the worst effect on mortar strength enhancement. The water absorption of nano-CuO mortar is the lowest because of the 60% reduction in capillarity values. All of the nanomaterials increased the workability, electrical resistivity and durability properties of specimens. Qing et al. [10] also reached a similar conclusion after adding equal amounts of CNTs and unequal amounts of nano-clay to a mortar. Morsy et al. [11] mixed different amounts of nano-clay into a mortar which contained CNTs, and found that it filled the gap of the cement mortar hardening the micro-structure framework, resulting in an increase in strength and density, which could increase the compressive strength by 18% at most. Arefi et al. [12] studied the influence of nano-alumina incorporation on the mechanical properties and microstructure of cement mortar, and found that it had a good effect on the enhancement of material strength when the dosage is 1% and 3%, because it could effectively fill the gaps between the cement matrix and reduce the number of calcium hydroxide crystals. Lee et al. [13] examined the effect of titanium dioxide (TiO_2_) nanoparticles on early aging and the long-term properties of cement-based materials through isothermal calorimetry, chemical shrinkage, setting time, compressive strength and surface microhardness, where TiO_2_ was used to replace part of the cement in the specimens. They found that early age hydration is accelerated by TiO_2_ nanoparticles. Compressive strength increases with higher TiO_2_ nanoparticle replacement at lower water-to-solids ratio (*w*/*s* = 0.40) and strength is not compromised by up to 10% TiO_2_ replacement at higher *w*/*s* 0.60. Senffa et al. [14] compared the photocatalytic activity and the rheological behavior of titania nano/microparticles (nT and mT) and zinc oxide microparticles (mZ) by adding them into cement paste in amounts ranging from 0 to 1.2 wt.%. The results showed that 0.67 mT:0.17 nT:0.17 mZ achieved the best and comparable performances in terms of photocatalytic activity. Samples with nT became less fluid and the additions had a little impact on the kinetics of hydration up to 120 min testing, but significantly for longer periods: mZ (delayed) and nT (shortened). I. Campillo et al. [15] determined the Vickers hardness of carbon nanotube cement-based composites under different curing time conditions, and analyzed the principles of mechanical reinforcement of carbon nanotubes in the cement matrix through bridging and other mechanisms. The results showed that the cement matrix and the carbon nanotubes have a good bonding effect. The single-walled carbon nanotubes and the multi-walled carbon nanotubes, respectively, increase the compressive strength of the cement hardened slurry after 14 d by 6% and 30%, and the toughness of the composite material shows a significant improvement. Onuaguluchi et al. [16] studied the effect of nanocellulose content on cement hydration and its mechanical properties. The results show that nanocellulose could effectively reduce the conductivity and delay the early hydration of cement, and the higher the dosage is, the more obvious the retardation effect is at 0%, 0.05%, 0.1%, 0.2% and 0.4% by mass of cement, but the cumulative heat of hydration and the degree of hydration are both higher than the blank group at 28 d of the age. Meanwhile, the mechanical properties are best with the content of 0.1 wt%. Compared with the blank group, the flexural strength of the specimen at 28 d is increased by 106%. With the increase of nano-cellulose content, the agglomeration of nanocellulose and strong combination of nanocellulose and cement paste, leads to a rise in the brittleness of the materials and a decline of mechanical properties. Flores et al. [17] also reached the same conclusion in their study. In addition, studies have shown that nanocellulose can also promote the hydration of older cements [18,19].

It can be seen that studies on single nanometer cement-based materials have been abundant, but it is rare to compare different types of nanomaterials under the same experimental conditions and evaluation indicators, which is not conducive to the performance comparison between different nanomaterials. In this paper, three kinds of nanomaterials such as nano-SiO_2_, CNTs and NCC were mixed with cement mortar, and the working properties such as compressive strength, flexural resistance and water absorption compared under the same conditions. Meanwhile, SEM, XRD and other microscopic methods were used to analyze the principles of action, and the comprehensive comparative analysis of strength benefit index was introduced to explore the influence of the three nanomaterials on the performance of cement-based materials.

## 2. Materials and Methodology

### 2.1. Raw Material

The raw materials used in this experiment include: C42.5 ordinary Portland cement, whose quality meets the requirements of the current national standard GB175-2007 “general Portland cement”; Ordinary river sand from Chongqing, in line with the standard JC/T622-2009 “silicate building products sand” specifications; The water used for mixing and maintenance is municipal tap water, which meets the technical specifications of JGJ63-2006 “concrete water standard”. Polycarboxylic acid type superplasticizer was produced by Shanghai Chenqi Chemical Technology Co Ltd. (Shanghai, China). CNTs dispersant was polyethylene pyrrolidone K30 which is from Shandong Yousuo Chemical Technology Co. Ltd. (Linyi, China). Technical indexes of the hydrophilic nano-SiO_2_, CNTs and NCC used are shown in Table 1.

### 2.2. Experiment Mixture Proportions 

The ratio of blank sample cement to sand was 1:3, the ratio of water to cement was 0.4, and the content of the water-reducing agent was 1% of the cement mass. In the experimental group, a certain amount of CNTs, NCC and nano-SiO_2_ were added on the basis of the blank group, and the specific dosage was shown in Table 2. 

In the table, 0.1%, 0.5% and 2% were the optimal dosage of three nano-materials in the other studies [16,20,21]. In order to meet the requirements of the content gradient, 0.01% and 3% dosage groups were added. The concentration of PVP (K30) is 0.6 g/L [22].

### 2.3. Specimen Preparation

When making nano-SiO_2_ test specimens, we first put the sand and cement into the mixer and stirred for 2 min. At the same time, the nano-SiO_2_ powder was added into half of the water requirement in a single portion and stirred evenly with a high-speed homogenizer at 2000 r/min for 8–10 min to mix it well. After the cement and sand are evenly mixed, the nano-SiO_2_ solution was slowly added, during which the mixer should not stop working. Then, the remaining half of the water is added in a beaker, washing the beaker and glass rod, and poured into the blender slowly again at a constant speed. After all the water is added, stirring is continued for 2 min to ensure even mixing. After stirring, the mortar was loaded into the mold in 3–4 portions, and a vibrating rod with a diameter of 2 cm was used for slight tamping after each loading. The time should be about 1 min to ensure the inner filling is tight. Then, the mold is gently vibrated for 20 times with both hands, left standing for 24 h, and the shape removed and put into a standard curing box, where it is maintained under the conditions of humidity above 95% and temperature set at (20 ± 2) °C.

The fabrication of CNTs and NCC specimens is slightly different from that of nano-SiO_2_. Since CNTs precipitate quickly in clean water, it is necessary to replace the fresh water with a 0.6 g/L PVP (K30) dispersion so that the CNTs are more uniformly dispersed in the aqueous solution, and because of the NCC colloid is difficult to disperse, it needs to be stirred at a rate of 2000 r/min for 2 min using a high-speed homogenizer to break up the bulk gel-like fiber, then the speed is increased step by step by 2000 r/min each time and stirred for 2 min. When the rate reaches 8000 r/min, the mixture is stirred for 2 min to disperse the fiber gel fully. The other steps are consistent with the preparation of the nano-SiO_2_ test piece.

### 2.4. Test Methods

#### 2.4.1. Compressive Strength

Specimens with a size of 70.7 mm × 70.7 mm × 70.7 mm were taken out after curing for 7 d and 28 d, respectively. A YAD-2000 microcomputer-controlled automatic pressure testing machine ( Changchun Kexin Test Instrument Co., Ltd., Changchun, China) was loaded at 1.0 KN/S speed until the test piece was destroyed and the average of three test pieces in each group was taken as the final test value of the group.

#### 2.4.2. Flexural Strength

The size of the test pieces is 40 mm × 40 mm × 160 mm, and they are taken out after curing for 7 d and 28 d, respectively. Then they are tested using a DKZ-5000 electric bending test machine (A YAD-2000 microcomputer-controlled automatic pressure testing machine ( Zhejiang Chenxin Machinery Equipment Co., Ltd., Hangzhou, China). The average value of three test pieces in each group is taken as the final value of the group test.

#### 2.4.3. Water Absorption Rate

The water absorption rate test is carried out according to the relevant specifications of “Testing Methods for Basic Performance of Building Mortar” (JGJ/T70-2009). The test piece size is 70.7 mm × 70.7 mm × 70.7 mm, and after curing for 28 days, it is placed at a drying box of 105 ± 5 °C, then weighed every 4 h until the difference between the two weighings is less than 0.1% of the mass of the test piece. It is then considered that the test piece has dried and the weight is recorded as the dry quality (m_0_). After cooling the test piece to room temperature, it is put it in a DK-8B electric heating constant temperature water tank ( Shanghai Jinghong Experimental Equipment Co., Ltd., Shanghai, China), and clean water injected until the water surface is about 20 mm higher than the test piece. After soaking for 48 h, the sample is taken out of the water, the excess moisture on the surface is wiped off with a wrung wet towel, and the sample is weighed. This weight is recorded as the water absorption mass (m_1_) of the test piece. The water absorption rate of the test piece is calculated as: w_x_ = [(m_1_ − m_0_)/m_0_] × 100%, and the average value of three test pieces in each group is taken as the final test value, and the result is accurate to 0.01%.

In the test data of compressive strength, flexural strength and water absorption, if the difference between the maximum or minimum values and the middle value exceeds 15% of the intermediate value, the maximum or minimum values should be eliminated, and the central value taken as the compressive strength value of this group. If the difference between the two measured values and the middle value both exceeds 15% of the intermediate value, the test results of this group are deemed invalid.

#### 2.4.4. SEM

SEM test instrument is a Sigma300 thermal field emission scanning electron microscope (Zeiss, Oberkochen, Germany). Firstly, the specimen was sprayed with gold on the surface of the sample to be scanned, and then amplified 5000–10,000 times to observe the cross-section microstructure of the test piece and take a picture.

#### 2.4.5. XRD

Samples were scanned with XRD-6100 instrument (Shimadzu, Kyoto, Japan) at a speed of 4°/min and a range of 10–80°.

## 3. Results and Discussion

### 3.1. Compressive and Flexural Strength

**Figure 1:**
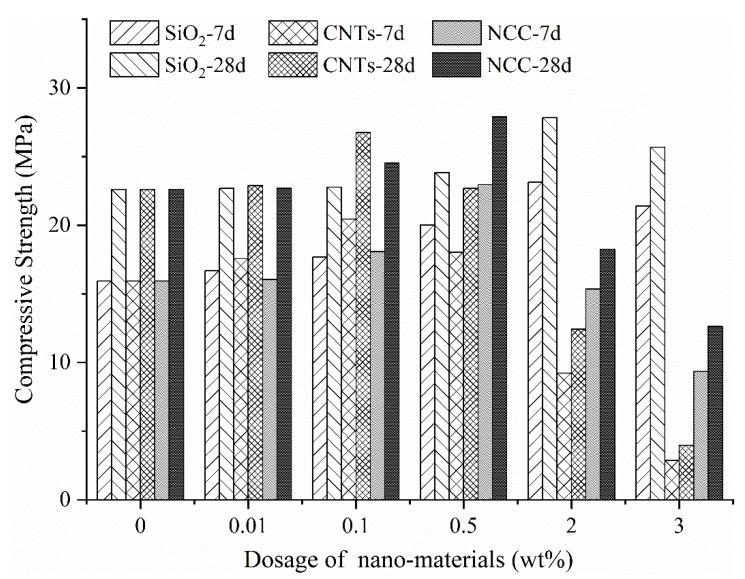
Effect of nanomaterial content on compressive strength.

Figure 1 shows the influence of the content of three nanomaterials on the compressive strength. From Figure 1, it can be found that the compressive strength of three nanomaterials at the age of 7 d and 28 d shows a downward trend after rising first, and their peak strength varies depending on the dosage. 

As a control, the compressive strength of the blank group is 15.93 Mpa at 7 d and 22.61 Mpa at 28 d. The peak value of compressive strength of the nano-SiO_2_ group appeared at 2%, which is 23.12 Mpa at 7 d and 27.83 Mpa at 28 d, an increase by 45.13% and 23.09% compared with the blank group. The optimum dosage of CNTs is 0.1%, and when the dosage of CNTs less than 0.1%, the compressive strength of the test piece increased with the amount of the CNTs. Conversely, the compressive strength decreased sharply. The compressive strength of the samples with 0.1% CNTs at 7 d and 28 d is 20.44 Mpa and 26.77 Mpa, which represent an increased by 28.31% and 18.40%, respectively, compared with the blank group. The peak values of compressive strength of the NCC group, which are 22.97 Mpa at 7 d and 27.9 Mpa at 28 d, appeared at 0.5%. Compared with the blank group, the compressive strengths at 7 d and 28 d increased by 44.19% and 23.40% respectively. In the case of their optimal dosage, the compressive strength of the nano-SiO_2_ and NCC groups were very close at the age of 7 d and 28 d, with a difference of only 0.15 Mpa at 7 d and 0.07 Mpa at 28 d, but both of them were higher than that of the CNTs group.

**Figure 2:**
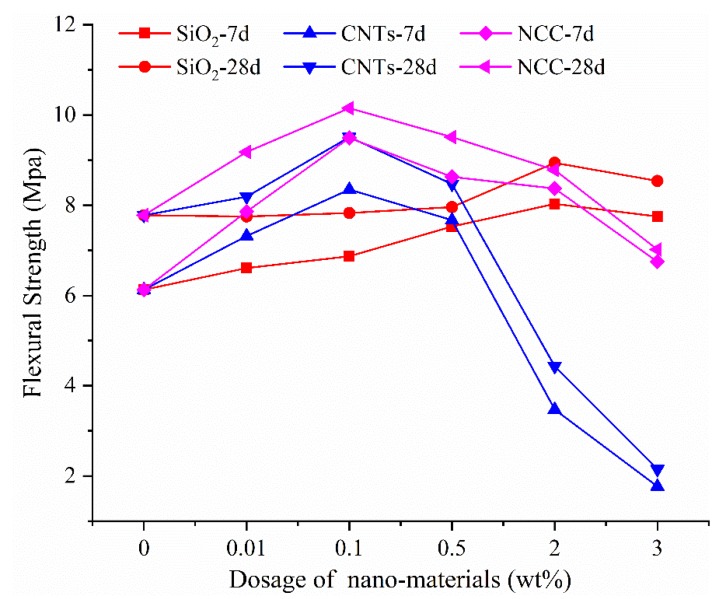
Effect of nanomaterial content on flexural strength.

Figure 2 shows the influence of the content on the flexural strength. Just like the trend of compressive strength with the dosage, all of them show a downward trend after rising first, and the the peak flexural strength also varies with the dosage. The peak value of flexural strength and the optimum dosage appeared at 2% for the nano-SiO_2_ group and 0.1% for the CNTs group. The peak value of flexural strength in the nano-SiO_2_ group is 8.03 Mpa at 7 d and 8.94 Mpa at 28 d, an increase by 31.00% and 14.91% compared with the values of the blank group, which are 6.13 Mpa at 7 d and 7.78 Mpa at 28 d. The peak values of the CNTs group are 8.35 Mpa at 7 d and 9.51 Mpa at 28 d, which represent an increase by 36.22% and 22.23% compared with the blank group. The significant difference with the other two materials is that the flexural strength is greatly affected by the dosage, so it can be seen in Figure 2 that the flexural strength decreased sharply when the dosage exceeded 0.1%. The optimum dosage of the NCC group is 0.1%, which gave a different from the compressive strength optimum value of 0.5%. The peak values of the NCC group are 9.49 Mpa at 7 d and 10.15 Mpa at 28 d, which is an increase of 54.81% and 30.46%, respectively, compared with the blank group. The highest flexural strength shows a decreasing trend of NCC > CNTs > nano-SiO_2_ at both 7 d and 28 d, and the intensity of the CNTs group changes with the dosage is larger than that of the nano-SiO_2_ and NCC groups.

**Figure 3:**
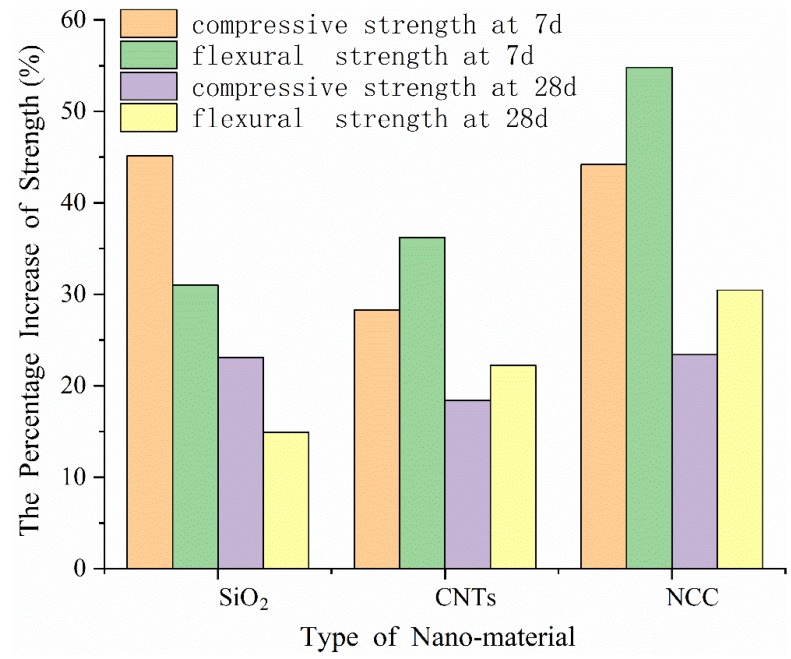
The percentage increases of the three nanomaterials at 7 d and 28 d.

Longitudinal comparison of the three nanomaterials in Figure 3 shows that all of them mainly act on the early strength improvement, and their effect on the 28 d compressive and flexural strengths is significantly lower than that at 7 d. However, there are differences among the three nanomaterials: the compressive strength percentage increase of the nano-SiO_2_ group was significantly higher than that of flexural strength, while CNTs and NCC groups showed higher increase percentages in the flexural strength.

It is not difficult to understand that this is related to the different micromorphologies and mechanisms of action of the three nanomaterials. Compared with CNTs and NCC, the circular granular morphology of nano-SiO_2_ is more conducive to filling the cement micropores which can effectively enhance the compactness of specimens, so it leads to a greater contribution to the compression. In addition, due to the shape of CNTs and NCC, they would bear a specific load in the process of specimen fracture and prevent the development of microcracks in the specimens, so their flexing resistance was improved more obviously.

### 3.2. Water Absorption

**Figure 4:**
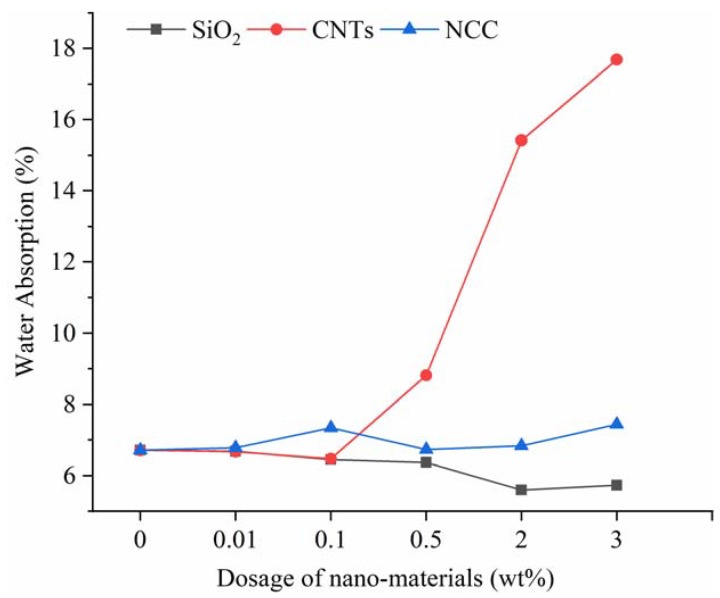
Effect of nanomaterial content on water absorption.

Water absorption tests were carried out following JGJ/T70-2009 “Standard test method for the basic performance of building mortar”. During the trials, the water absorption of the specimen at 48 h was recorded and calculated, as shown in Figure 4.

Figure 4 shows that the three nanomaterials are beneficial to reducing the water absorption rate, but the trend of water absorption rate with the amount of nanomaterial is varied. The lowest water absorption rate of nano-SiO_2_ appeared for the 2% dosage group, just same as the strength, but it decreased first and then increased slowly with the dosage, which is contrary to the strength trend. This phenomenon shows that the internal micropores of the pieces are indeed adequately filled by nanomaterials, leading to the compactness increasing gradually, and hence to the increase in strength and decrease in water absorption. The internal agglomeration phenomenon is severe in the case of 3% loading because of the excessive content, and the filling effect of micropores is worse than at 2%. Meanwhile, coupled with the water absorption of nano-SiO_2_ itself, the water absorption rate of 3% is slightly higher than that of 2% content.

The water absorption rate ranking of the CNTs group is 0.1% < 0.01% < 0% < 0.5% < 2% < 3%, an overall trend which is the opposite of the compressive strength one, indicating that CNTs also have a good filling effect on the internal pores of mortar specimens. However, with the increase of the amount of CNTs, a complex network structure is formed inside the mortar, that makes the cement hydration products unable to be combine tightly, and leads to an increase of pores. Due to the strong water absorption of CNTs, the two effects result in the water absorption of the 0.5%, 2% and 3% groups increasing rapidly and being much higher than in the blank group.

The water absorption rate ranking of the NCC group is 0% < 0.5% < 0.01% < 2% < 0.1% < 3%. Different from the other two nanomaterials, they show a tendency to rise first, then decrease and then rise again, which has no significant relationship with the strength trend. There are mainly two reasons for this phenomenon, on the one hand, the 0.01% and 0.1% dosing group have a limited contribution to the compactness of the test piece. On the other hand, the NCC’s water absorption is strong, so its water absorption is higher than the blockage of water formed by the filling of the micropores. Due to the excessive addition, the 2% and 3% dosage groups formed a complex interlaced hollow network structure, and at the same time, combined with the water absorption of the fiber, thus causing excessive water absorption. However, the water absorption rate of the 0.5% dosage group is the lowest and very close to the blank group, indicating that the amount of water absorbed by NCCs and the amount of water retained by the NCC filling effect reached an equilibrium state.

The longitudinal comparison of water absorption of the three nanomaterials shows that the water absorption of CNTs and NCC is higher than that of the nano-SiO_2_ group as a whole. The minimum water absorption of the nano-SiO_2_ group was at least 15.54% lower than the minimum value of other two nanomaterials, meaning that the filling effect of nano-SiO_2_ on the micro-pores is better than that of CNTs and NCC, which is mainly due to the fact the granular shape of SiO_2_ is more suitable for the filling of pores than the long strip-like forms of CNTs and NCC.

### 3.3. Microstructure Analysis

The improvement of the macromechanical properties of cement-based materials by nano-materials mainly results from their excellent microstructure and effect. By observing the micro-morphology characteristics of the failure surface of the specimens, the distribution of nanomaterials in the cement-based materials can be analyzed, especially the morphology of the long strip-like CNTs and NCC nano-materials after the sample is damaged under compression, so that the mechanism whereby the three nanomaterials improve the mechanical properties of cement-based materials can be analyzed further.

**Figure 5:**
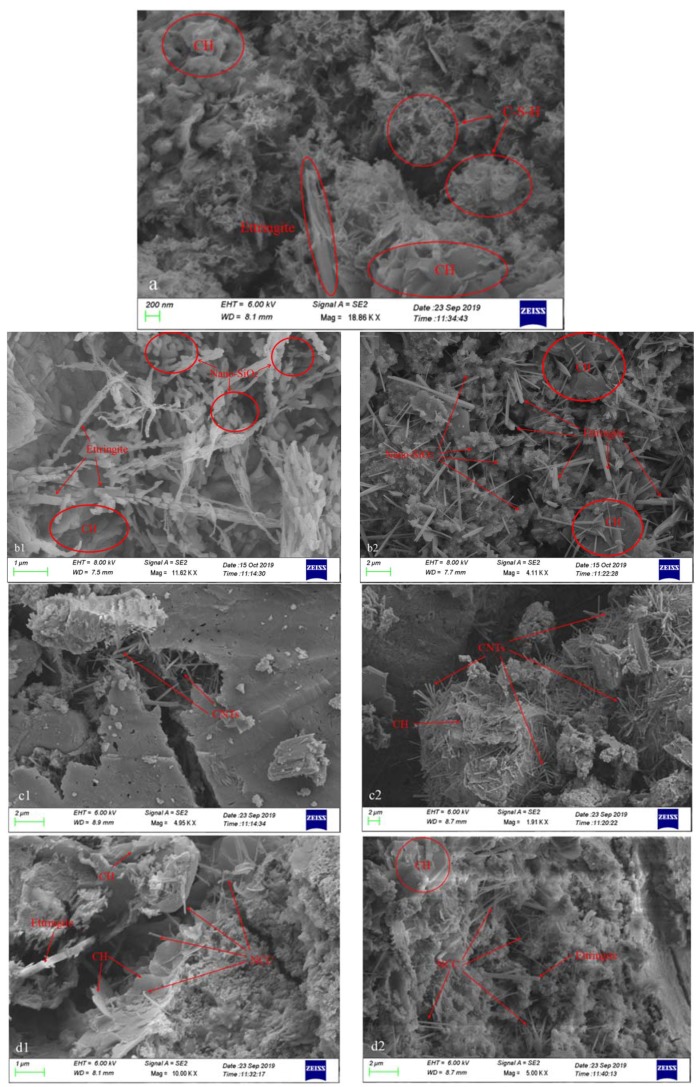
SEM micrographs of cement mortar failure surface at age of 28 d: (**a**) blank group; (**b1**) 2% nano-SiO_2_; (**b2**) 3% nano-SiO_2_; (**c1**) 0.1% CNTs; (**c2**) 3% CNTs; (**d1**) 0.5% NCC; (**d2**) 3% NCC.

Electron microscopy was performed on the 28 d blank group, 2% and 3% nano-SiO_2_ group, 0.1% and 3% CNTs group, and 0.5% and 3% NCC group. The scanning results are shown in Figure 5. As can be seen from Figure 5a, the section of the blank group was relatively loose and porous. Even if enlarged to the size of 200 nm, there was still no connection or filler in it, which was different from the specimens mixed with nanomaterials.

In the graph, when the content is 2%, the nano-SiO_2_ particles adhere to the ettringite as a micro-aggregate, which plays a crucial filling role for the micropores. At the same time, it can be seen from the picture scale that its particle size is larger than that of a single SiO_2_ particle, indicating that nano-materials play a role of crystal nucleus in the cement hydration process. The continuous hydration of cement on its surface makes the nano-SiO_2_ particles continuously increase, and its compactness is significantly better than that of the blank group. However, when the content increased to 3%, this excessive content led to insufficient dispersion of SiO_2_ particles, which mainly exist in the form of an agglomerated state, and the size even reached the micron level. The filling effect of the internal nano-pores was weak, and strength loss points were formed inside the mortar, which caused a negative impact on the strength of the mortar, and the density was also less than that of the group with 2% content.

Figure 5(c1,c2) are SEM images of the CNTs group. They show that when the dosage is appropriate (Figure 5(c1)), the crack expansion is hindered by the CNTs, and the cracks can develop only when the CNTs are broken, indicating that the CNTs mainly withstand the tensile stress and have the effects of bridging, pulling out and strengthening the test piece [23,24], so that the compressive and flexural strength of the test piece is improved. In Figure 5(c2), the CNTs are excessively mixed, forming a complex and intertwined CNTs network. The cement hydration product C-S-H cannot enter the interlaced CNTs network completely, instead, increasing the pores inside the mortar specimen and leading to a significant reduction in strength. The same phenomenon appears in the SEM image of the NCC. As shown in Figure 5(d1), plant fibers which are obviously broken are visible at the cracks, and the same as the CNTs, mainly by taking a specific load to prevent the crack from continuing to develop, thereby improving the strength. However, similarly, the incorporation of an excessive amount of NCC forms a complex network structure and agglomeration to form inside the mortar, which hinders the filling of the micro-pores by the cement hydration product. Also, such a porous network structure causes the cement hydration products to not be intimately bonded, forming a flocculated porous structure as shown in Figure 5(d2), resulting in a decrease in strength.

### 3.4. XRD Phase Analysis

**Figure 6:**
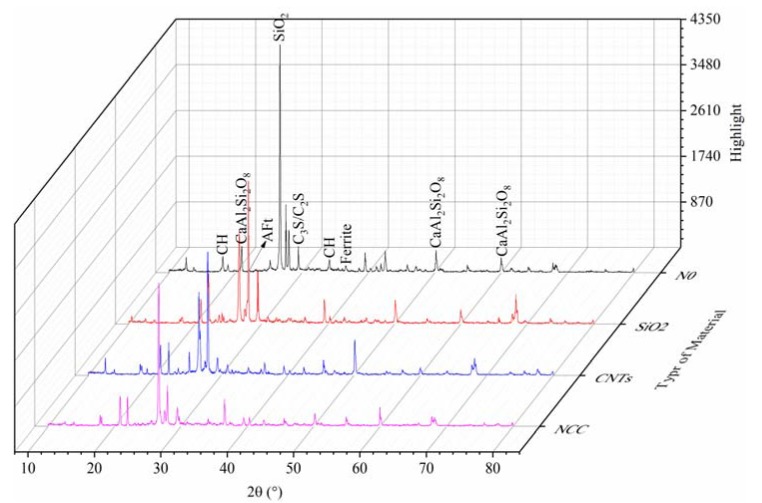
*X*-ray diffraction patterns at 28 d of age.

At a rate of 4 °/min and a range of 10–80°, 28 d-old specimens of the blank and the three kinds of nanomaterials groups were scanned, and the *X*-ray diffraction analysis results are shown in Figure 6. 

Different phases were identified according to different diffraction angles, and according to the characteristic peaks of each phase, the semi-quantitative method was used to analyze and compare the number of corresponding substances. After peak fitting, it was determined that the characteristic peak of SiO_2_ is at 26.64°, the characteristic peak of cement mineral C3S is at 29.4°, the characteristic peak of the hydration product Ca(OH)_2_ is at 18.1° and the characteristic peak of the hydrated product Aft is at 22.1°. The specific peak values are listed in Table 3.

Figure 6 and Table 3 present the XRD patterns and characteristic peaks of the different nanomaterial specimens. It can be seen from Figure 6 that there no new particular diffraction peaks were formed after the addition of the nanomaterials, indicating that the addition of the nano-materials did not produce new substances different from the blank group. Then we analyzed the increase and decrease of hydration reaction materials of the cements. The first is the content of C3S and SiO_2_ in the cement hydration raw materials. It can be seen from Table 3 that the content of SiO_2_ and C3S in sand and cement is significantly reduced after the incorporation of the three kinds of nanomaterials, proving that the addition of the three nanomaterials promoted the hydration balance to move to the right, which promotes in turn the hydration of cement. The increase of cement hydration products increases the compactness of the test piece and improves the strength of the test piece.

Considering the values of the hydration products CH and AFt, which can be obtained from Table 3, after the addition of the nanomaterials, the CH peak of the hydration products decreases, and the CH of the SiO_2_ group is relatively the least. The reason is that in the later stages of cement hydration, the nanomaterial will react with the cement hydration product CH further or promote the later hydration of the cement, so that the CH of the nanodoped material group is reduced compared with the blank group. Among the three nanomaterials, the reactivity of nano-SiO_2_ is the highest, so the peak of CH is less than CNTs and NCC [22,25,26]. Aft does not react with nanomaterials, and the accumulation effect makes the amount of increase much more significant than for CH, and the Aft stacks the most in the nano-SiO_2_ group, which is related to promoted hydration balanced re-movement.

The crystal orientation of CH has an essential influence on the strength of cement-based materials. The diffraction peaks of CH can determine it at two angles in XRD. The crystal orientation of CH can be obtained by comparing the peak intensities of crystal faces in different crystal directions [10]. The CH crystal orientation index R is calculated as follows [27]:

(1) $$R=\frac{1}{0.74}\frac{I_{001}}{I_{101}}$$

where $I_{001}$ and $I_{101}$ are the peak strengths of the crystal plane at CH/(001) and CH/(101) in Table 3. When the arrangement of CH is not oriented, R = 1; When the arrangement of CH has a tendency, R > 1, and the larger R is, the stronger orientation is, and the more serious the direction of CH is, that is, the closer the direction is, the lower the strength is. The CH orientation index of each group is shown in Table 4. It can be seen from Table 4 that after the addition of the nanomaterials, the R values of the three materials are all reduced, indicating that the three nanomaterials reduce the degree of orientation of the CH crystals to a certain extent, preventing the growth of the CH crystals, and causing a refinement, and this also explains the principle of how the three nanomaterials enhance the strength of cement-based materials.

### 3.5. Strength Benefit Analysis

All three nanomaterials are industrially produced, so the cost of large-scale application is one of the primary considerations. Taking the three kinds of nanomaterials in this experiment as examples, the average price per kg of each material was obtained through inquiries of the three manufacturers. Then the dosage and price of each nanomaterial required for 1 m^3^ cubed mortar were calculated based on the dosage of a standard compressive specimen (70.7×70.7×70.7). The specific calculation process is as follows: according to the actual test, the cement content of a single sample is about 190 g, based on the mass of the cement of a single test piece, multiplied by the optimum amount, which is the amount of nanomaterial in a single test piece, and multiplied by the price, the cost of the nano-materials of a single test piece can be obtained, so the value of the nanomaterial added to cement mortar per m^3^ could be calculated finally.

Both strength and economic benefits will affect peoples’ choice of nanomaterials, and the weights of the two are different for different users and diverse application scenarios. Therefore, to more intuitively and objectively reflect the economics of the three nanomaterials, it is proposed to evaluate the economic efficiency, that is the strength benefit, by using the cost of per 1% strength promotion, using the following formula:

(2) $$B_{s}=\frac{C}{P}$$

In the formula, Bs is the strength benefit, C is the nanomaterial cost required per unit cubic meter of cement mortar, and P is the percentage increase of the strength of the nanomaterial, so the lower the Bs value is, the more economical the nanomaterial-modified material is.

**Figure 7:**
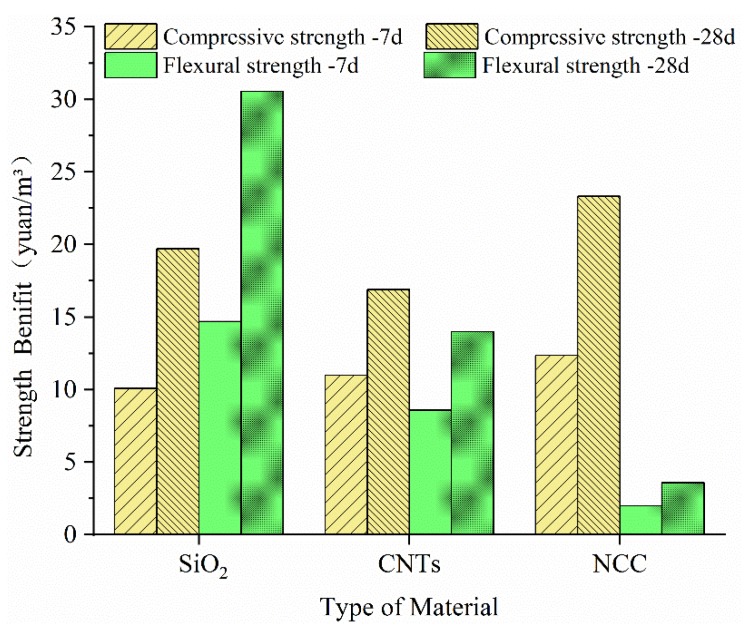
Compressive and flexural strength benefit of three nanomaterials.

Combined with the compressive strength, flexural strength and the cost of increasing 1% strength unit cube calculated by the above formula, the final strength gain is shown in Figure 7.

From the compressive strength benefits, for every 1% increase in strength, the lowest cost at 7 d is for the nano-SiO_2_ group – 10.09 yuan, and the lowest at 28 d is for the CNTs group – 16.91 yuan. The reason why nano-SiO_2_’s 7 d strength benefit is the lowest is that although its price is only 1/5 that of other two nanomaterials, on the other hand, its effective content is the highest of three nanomaterials, so its strength benefits are lower than those of the other two nanomaterials but not obviously. The CNTs are the most expensive nanomaterial, but thanks to the minimum amount used, its 28 d strength benefit is the lowest, while NCC’s 7 d and 28 d strength benefits are the highest of them but the most uneconomical because of its higher price, similar strength increase to nano-SiO_2_ and moderate dosage. The flexural strength benefit is very obvious: whether at 7 d or 28 d, it shows the trend of nano-SiO_2_ > CNTs > NCC. Because the cost of nanomaterials per m^3^ mortar is nano-SiO_2_ > CNTs > NCC, and the percentage of flexural strength improvement is nano-SiO_2_ < CNTs < NCC, the strength benefits of the three nanomaterials are significantly reduced gradually. Overall, CNTs had the lowest compressive strength benefit, while NCC had the lowest flexural strength benefit. In practical application, nanomaterials can be selected according to the actual needs.

## 4. Conclusions

In this paper, nano-SiO_2_, CNTs and NCC were used as raw materials to prepare cement-based composites to compare the effects of the three nanomaterials on the mechanical and water absorption properties. The main conclusions are as follows:

- The three nano-materials significantly improved the mechanical properties of cement-based materials. The 7 d compressive strength of SiO_2_, CNTs and NCC increased by 45.13%, 28.31% and 44.19%, and the 28 d compressive strength increased by 23.09%, 18.40% and 23.40%, respectively. The 7d flexural strength increased by 31.00%, 36.22% and 54.81%, and the 28d flexural strength increased by 14.91%, 22.23% and 30.46%, respectively. Overall, NCC has the best strength enhancement effect on cement-based materials.
- Among the three kinds of nanomaterial, SiO_2_ reduces the water absorption of test pieces due to its effective micro-porosity filling effect. The other two have a limited filling impact on the micro-pores due to their micro-morphology, and the materials themselves have a stronger water absorption, resulting in different degrees of improvement of the water absorption, especially CNTs, which water absorption rate reached nearly three times that of the blank group.
- Through SEM and XRD analysis, it was found that the three nanomaterials all promoted cement hydration, and also reduced the crystal orientation of the cement hydration product CH, but the specific principles of the strength improvements were different. Nano-SiO_2_ mainly makes the cement hydration more complete by acting as a crystal nucleus, and fills the internal micropores of cement-based materials to increase the compactness of materials and thus improve their mechanical properties. However, CNTs and NCC overlap with each other in cement-based materials, and bear a specific load through bridging action and the high elastic modulus of the nanomaterials themselves, while at the same time, by dispersing and changing the load transfer path to prevent the development of cracks they can effectively improve the mechanical properties of materials, especially the flexural strength.
- Through economic analysis and taking strength benefit as the evaluation index, it can be known that CNTs has the highest cost-performance ratio as far as compressive resistance is concerned, while for flexural resistance NCC has the highest cost-performance ratio. In practical applications, nanomaterials can be selected according to the different desired weights of compression and bending resistance.

## Figures and Tables

**Table 1:** Technical indexes of the nanomaterials.

Material	Properties
Nano-SiO_2_	Purchased from Beijing Shenghe Haoyuan Technology Co. Ltd. (Beijing, China); white powder, SiO_2_ content not less than 99.5%, particle size is 20–30 nm, specific surface area not less than 250 m^2^/g
CNTs	Purchased from Suzhou Tanfeng Graphene Technology Co. Ltd. (Suzhou, China); black lightweight powder, inner diameter is 3–5 nm, outer diameter is 8–15 nm, length is 3–12 microns, specific surface area not less than 233 m^2^/g, C > 95%, ash content C < 3%, volume density 0.15 g/cm^3^
NCC	Purchased from Zhongshan NFC Bio-materials Co. Ltd. (Zhongshan, China); transparent gel with a concentration of 2.5% ± 0.5%, a width of 5–100 nm, a length of > 1 μm, an elastic modulus of 6.2–6.9 GPa, and a tensile strength of 222–233 MPa

**Table 2:** The dosage of nano-materials.

Materials	Dosage (wt% of Cement)				
**CNTs**	0.01	0.1	0.5	2	3
NCC	0.01	0.1	0.5	2	3
Nano-SiO_2_	0.01	0.1	0.5	2	3

**Table 3:** Diffraction peaks of cement hydration raw materials and products at 28 d of age.

Sample	Crystal					
	CH/(001)	CH/(101)	CaAl_2_Si_2_O_8_·4H_2_O	AFt	SiO_2_	C3S
NO	324	264	516	68	4354	1306
Nano-SiO_2_	144	125	486	966	1814	294
CNTs	194	165	578	636	1598	284
NCC	188	160	584	570	2744	312

**Table 4:** CH crystal orientations of cement reinforced by three nanomaterials at 28 d of age.

Sample	CH/(001	CH/(101)	Orientation of CH Crystal
N0	324	264	1.657
SiO_2_	144	125	1.555
CNTs	194	165	1.587
NCC	188	160	1.586
